# 3D Printing and Bioprinting Nerve Conduits for Neural Tissue Engineering

**DOI:** 10.3390/polym12081637

**Published:** 2020-07-23

**Authors:** Xiaoling Yu, Tian Zhang, Yuan Li

**Affiliations:** 1 School of Chemistry, Chemical Engineering and Life Science, Wuhan University of Technology, Wuhan 430070, China; yxlhend@whut.edu.cn; 2 State Key Laboratory of Silicate Materials for Architectures, Wuhan University of Technology, Wuhan 430070, China

**Keywords:** 3D printing, bioprinting, hydrogel, polyester, peripheral nerve regeneration

## Abstract

Fabrication of nerve conduits for perfectly repairing or replacing damaged peripheral nerve is an urgent demand worldwide, but it is also a formidable clinical challenge. In the last decade, with the rapid development of manufacture technologies, 3D printing and bioprinting have been becoming remarkable stars in the field of neural engineering. In this review, we explore that the biomaterial inks (hydrogels, thermoplastic, and thermoset polyesters and composite) and bioinks have been selected for 3D printing and bioprinting of peripheral nerve conduits. This review covers 3D manufacturing technologies, including extrusion printing, inkjet printing, stereolithography, and bioprinting with inclusion of cells, bioactive molecules, and drugs. Finally, an outlook on the future directions of 3D printing and 4D printing in customizable nerve therapies is presented.

## 1. Introduction

**Figure 1:**
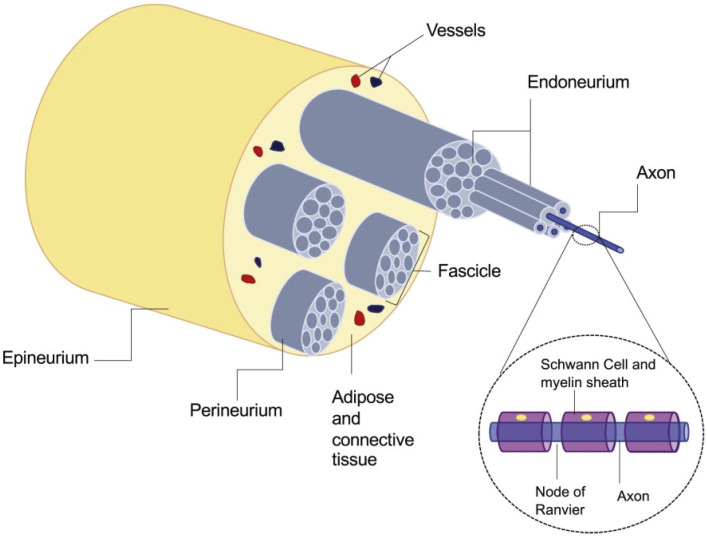
Normal peripheral nerve anatomy. Reproduced with permission [4]. Copyright 2020, Elsevier Ltd.

Peripheral nerve injury is a very common neurological disease that is generally caused by direct mechanical trauma [1]. Although peripheral nerve injury is not life-threatening, it will lead to a significant decline in the quality of daily life, including sensory and motor dysfunction, nerve palsy, muscle atrophy, etc. which annually affects more than one million people worldwide [2]. In addition, the self-repairing ability of peripheral nerves is very limited, which is related to the patient’s age and injury mechanism, especially to the closeness of the injury to nerve cells [3]. The normal peripheral nerve anatomy view is shown in Figure 1. After the peripheral nerve ruptures, both distal stumps are retracted separately. The distal stump loses the supply of nutrients from the neuron cell body, so that the protrusions and myelin sheaths degenerate and eventually disappear completely, while the proximal degeneration may be restored.

If the operation can be carried out in time to suture the broken end to the right, it will help to promote axon regeneration, and accurately reach the end to restore nerve function [5]. At present, the clinical treatment method is to carry out delicate surgical suture by tension-free neural tube suture. However, the suture repairing cannot be conducted if the length is over 5 mm. In this case, autologous nerve transplantation remains the gold standard, but this method requires at least two surgeries at the expense of healthy nerves and may cause neuroma formation, so the supply of donor nerves is limited [6]. According to the above analysis, there are some limitations in both surgical suturing and autogenous nerve transplantation. Therefore, people search for solutions through neural tissue engineering which provides exogenous substitutes to bridge the nerve stumps.

Tissue engineering aims to develop replacements for functionally impaired tissues and organs [7]. It usually requires a scaffold to provide transitional three-dimensional support for cell attachment, migration, and proliferation, as well as a carrier for transporting biologics [8,9]. Moreover, nerve tissue engineering scaffold can assist the nerve reconstruction in long distance nerve defects, which can reach length of 4 cm or even longer and it provides high accuracy and microarchitecture [10].

Rapid prototyping technologies, 3D printing and bioprinting have become remarkable in the field of tissue engineering. Based on digital model files, 3D printing is a technology that uses adhesive materials to construct objects by layer-by-layer printing [11]. The rapid development of 3D printing technology has accelerated the arrival of personalized medicine era. With the development of various printing methods and materials, 3D printing technology is widely used in medical applications, including rebuilding tissues/organs for clinical treatment or building tissues in disease models [12,13]. So far, many types of biomimetic tissues have been created through 3D printing, such as bone tissue [14], cardiovascular tissue [15], nerve tissue [16], skin tissue [17], muscle tissue [18], etc. The main advantages of 3D printing are personalized design and precise manufacturing. To be specific, (1) the accuracy of machine-based manufacturing is much higher than that of manual manufacturing, and it can reproduce the entity accurately with micron-scale resolution, (2) in combination with 3D imaging technology, 3D printing can quickly and accurately manufacture personalized tissue engineering scaffolds based on the imaging data of the patient’s defect/lesion site to achieve a perfect match between the stent and the patient’s defect/lesion site, and imitate the microstructure of natural tissue in morphology, (3) 3D bioprinting can even print materials, cells, proteins and other biologics together, and promote the growth and differentiation of cells on scaffolds by controlling the arrangement of cells to obtain the desired tissue repair effect [19]. Therefore, 3D printing technology has great advantages in the construction of peripheral neural scaffolds.

In this review, we introduce various 3D printing technologies that have been used in nerve guidance conduit manufacturing and we will summarize the recent advancements and future perspectives for materials that are suitable for these 3D printing/bioprinting in this biomedical field. Firstly, we discuss the requirements for an ideal peripheral neural scaffold. Secondly, we introduce the 3D printing technologies applied in neural tissue engineering. Thirdly, we summarize materials that have been developed for 3D printed neural scaffolds, including natural materials, synthetic materials and composite materials, especially hydrogels, thermoplastic and thermoset polyesters and conductive composite [20,21,22]. Furthermore, 3D bioprinting technologies widely used in neural scaffolds are described, and utilized bioink components materials, cells, and bioactive molecules are also reviewed [23]. Finally, a prospect on the future research directions of emerging 3D printing technologies and stimulus-responsive 4D printing is provided [24].

## 2. Requirements for Ideal Peripheral Neural Scaffolds

The ideal neural tissue engineering scaffold can mimic the composition and structure of the extracellular matrix (ECM) to facilitate cell seeding, adhesion, proliferation, differentiation, and tissue generation [25,26]. In addition, micro grooves also play a guiding role in the directional growth of nerves, because the correct reconnection of nerve bundles helps to restore nerve function [27]. Zhu et al. [28] printed a four-microchannels nerve conduit with a complex structure. After implanting the 3D printed conduit between the transected sciatic nerves of the rat, it was observed that the regenerated nerve branched into four branches at the proximal end and merged into a nerve at the distal end, and the injured nerves showed promising recovery. This suggests that the guiding fascicular structure can simulate the direction and track of nerve growth [29,30]. In the actual situation, the structure of the scaffold should be designed according to the injury of the native nerve, so as to prevent the nerve incorrect connection and play a correct guiding role on the nerve stump. The high resolution of 3D printing technology can easily achieve the accuracy and complexity required for neural scaffolds.

Neural tissue scaffolds need to replicate the mechanical properties of local nerves, and different materials exhibit different tensile strengths. It is known from the literature that the maximum tensile strength of normal sciatic nerve and acellular nerve in rats is 2.7 and 1.4 MPa [31]. Therefore, if nerve scaffolds are to be implanted into rats for animal experiments, the mechanical properties should reach the corresponding level. Similarly, if a scaffold is to be implanted in a patient, it must first protect delicate nerves, and its ability to withstand stretching, compression, and shearing forces needs to be tested [32].

Neural scaffolds should be biocompatible and have very low cytotoxicity and inflammatory response in the body. They should provide good cell anchoring site, which is conducive to cell adhesion, growth and proliferation, and normal expression, so as to promote the reconstruction and functional recovery of injured nerves [33]. This depends on the selection of materials and bioactive molecules for neural tissue engineering. Furthermore, one of the main functions of the nervous system is the transmission of electrical signals, which means that an ideal nerve scaffold, in addition to providing a physical support for cells, would be better to have electrical properties that enhance the proliferation and migration of nerve cells to promote nerve regeneration [34].

## 3. 3D Printing Technologies for Nerve Regeneration

3D printing represents a series of flexible additive manufacturing technologies that can accurately construct structures with complex 3D features. It has great advantages in design flexibility, mass customization, reliability, and diversity of compatible materials [35]. The principle of the 3D printing method is to use a computer-controlled stage and cured printed materials guided by predefined digital models to build a 3D structure layer by layer. 3D printing technology has appeared in the last century and has developed into a variety of printing methods such as extrusion, inkjet, and stereolithography (SLA). For more details, we refer the readers to some excellent reviews [35,36]. This section is a summary of the 3D printing methods widely used in neural tissue engineering, and the prospect of the latest 3D printing methods.

### 3.1. Basic 3D Printing Technologies

**Figure 2:**
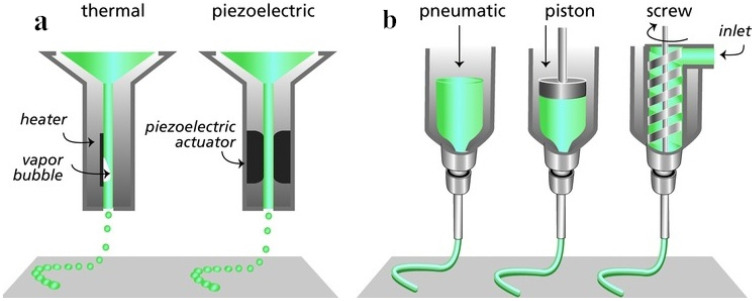
Schematic illustration of two 3D printing technologies according to working principles. (**a**) inkjet printing (thermal, piezoelectric); (**b**) extrusion printing (pneumatic, piston, screw). Reproduced with permission [40]. Copyright 2013, WILEY-VCH Verlag GmbH & Co. KGaA, Weinheim.

Inkjet printing is a 3D manufacturing technique that can deposit tiny droplets of polymer solution along the x, y and z axes in a highly controlled manner, thus repeatedly producing patterns on the substrate (Figure 2a). The ink material must remain in liquid form in order to be able to form droplets and solidify immediately upon deposition to build a 3D structured material stack [37]. Compared with other printing methods, the deposition volume of inkjet printing is smaller so as to achieve better printing resolution [27]. Delia et al. [38] dissolved polylactide polycaprolactone at a concentration of 1% to 10% *w/v* in various solvents (such as cellosolve acetate and chlorinated organic compounds), and installed it into the injection device to identify stable injection parameters for the manufacture of 1 μm resolution nerve conduits.

Extrusion printing is the layer-by-layer deposition of writing through the movable nozzle of the extrusion print head under computer control (Figure 2b). It is divided into fusion-based processes (e.g., fused deposition modeling (FDM)) and dissolution-based processes (e.g., 3D plotting). Generally the writing materials are molten/semi-melted polymers, polymer solutions, pastes or dispersions [21], most of which are viscous materials. Hsiao et al. [39] extruded 195 °C molten polylactic acid through a 0.25 mm copper extrusion nozzle to obtain a 3D scaffold. The variability of the scaffold structure can be achieved by FDM, and its micro-level or even nano-scale porous isotropic fibers to induce human dental pulp stem cells show directionality and neural differentiation.

**Figure 3:**
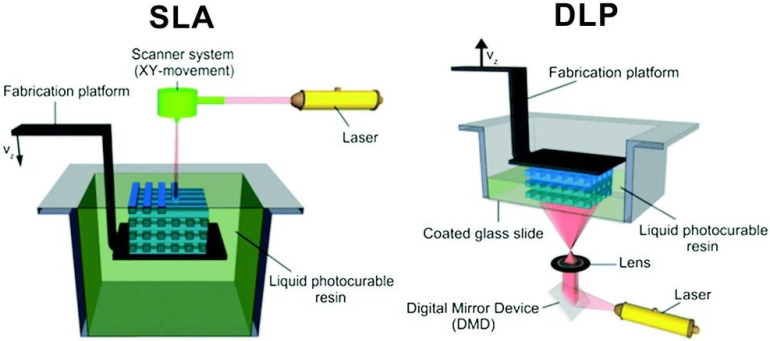
Scheme of bottom-up stereolithography (SLA) and top-down digital light processing (DLP) setups. Reproduced with permission [42]. Copyright 2012, Elsevier Ltd.

Stereolithography process is based on the photopolymerization principle of liquid photosensitive resin [41]. When the laser beam controlled by the computer tracks the resin liquid surface under the action of the deflection mirror, the liquid is solidified from point to surface where the light spot is scanned. After the first layer scan is completed, the lifting platform drives the platform to descend one layer-height, and then scans the next layer until it gets a complete 3D solid scaffold (Figure 3). SLA is a relatively slow and discontinuous printing process, and the requirements for materials are photosensitive viscous polymers [35].

Digital light processing (DLP) technology is also based on the resin photopolymerization [43]. Based on the same principle, the main difference between the DLP and SLA methods is that the SLA projects the light spot of the laser beam, and the digital micro-mirror device (DMD) in the DLP consisting of millions of mirrors can directly project a 2D image onto the photosensitive material flat, which greatly improves the printing efficiency (Figure 3) [42]. Zhu et al. [28] constructed the GelMA-PEGDA nerve conduit with four microchannels by DLP. The Young’s modulus of the conduits under different printing conditions are between 6.7 and 16.6 mW/cm^2^, and can be printed within 2 to 10 min. The 3D printed conduit was implanted between the proximal and distal ends of the sciatic nerve in the transected mice. After 11 weeks of implantation, the regenerated nerve branched into four branches at the proximal end and fused into one nerve at the distal end. The injured sciatic nerve of the rat also recovered motor and sensory functions after transplantation. Ye et al. [44] also printed the GelMA multi-channel neural conduit through DLP with optimized printing parameters, and co-cultured the neural conduit with PC12 cells in vitro, confirming that the neural cells can undergo longitudinal proliferation and migration with the support of multiple channels, and neural crest stem cells are induced to differentiate into neurons in the neural conduit.

### 3.2. Emerging 3D Printing Technologies

**Figure 4:**
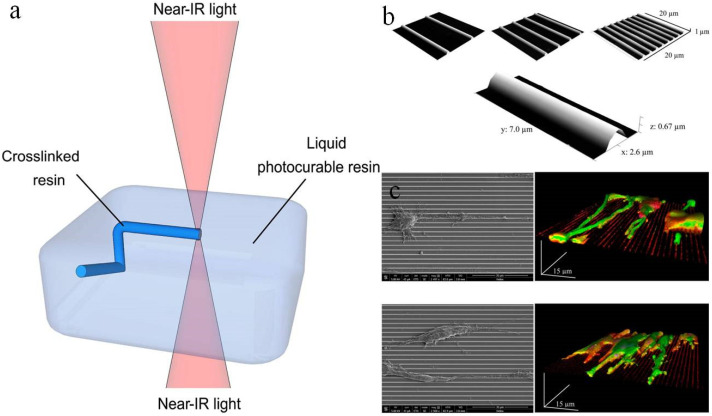
(**a**) Schematic illustration of two-photon polymerization (TPP). Reproduced with permission [42]. Copyright 2012, Elsevier Ltd. (**b**) SEM images of the patterned surfaces by TTP. Reproduced with permission [52]. Copyright 2013, American Chemical Society. (**c**) SEM images (on the left) and 3D rendering of a z-stack confocal acquisition (on the right) of PC12 and SH-SY5Y cells. Reproduced with permission [52]. Copyright 2013, American Chemical Society.

The two-photon polymerization (TPP) method was developed by Japanese researchers in the early 21th century [45,46]. The TPP refers to the photopolymerization process initiated by two-photon absorption (one molecule of a substance absorbs two photons at the same time) at the high-intensity laser focal point generated by the near-infrared femtosecond pulsed laser beam. Two-photon absorption does not occur in the optical path except for the focal point (Figure 4a). Therefore, compared with the traditional SLA method, the TPP method jumps out of the limitations of the surface and can directly perform photopolymerization at any point in the three-dimensional space [47,48]. Moreover, the most notable feature of this method is that it has potential solidification resolutions below the diffraction limit of the applied light, which enables the 3D printing to reach the micro-nano scale and the lateral spatial resolution to reach 80 nm [49]. However, high resolution also brings a slow printing process, which is difficult to use in industrial manufacturing, but suitable for areas where structural requirements are more delicate, such as tissue engineering.

Koroleva et al. [50] printed neural scaffold by combining TPP and mold imprinting soft lithography technology, which achieved high resolution while greatly increasing the printing speed. TPP is used to manufacture the prototype scaffold structure using photopolymerizable polylactic acid. The prototype scaffold is stamped by soft lithography PDMS to obtain the mold, and then the PDMS mold is used to generate multiple copies of the same scaffold through micro-molding technology. The TPP process takes about 3 to 5 h, while imprinted soft lithography takes only 10 min. The 3D scaffold provides a suitable matrix to support Schwann cell adhesion, so that cells are observed to form bipolar and tripolar morphologies. Angelo et al. [51] reported on the development process of the 3D scaffold made of PEGDA. The high resolution of the two-photon polymerization method can create a truly prominent structure. This 3D scaffold can support the growth and proliferation of neuro 2A cells and induce the formation of nerve filaments and nerve extension. Marino et al. [52] created a submicron patterned scaffold by direct laser writing based on TPP, and studied the effect of this scaffold on cell morphology. The results showed that both of rat PC12 neuron-like cells and human SH-SY5Y-derived neurons differentiated into tightly arranged and significantly longer neurites on parallel aligned submicron bumps. Morphological characterization by scanning electron microscopy revealed the scaffold structure and cell arrangement on the scaffold (Figure 4b,c).

Continuous liquid interface production (CLIP) is a patented technology proposed in 2014 [53], This method uses ultraviolet rays to cure the photosensitive resin while pulling the product out of the resin bath. Compared with the traditional SLA and DLP that rely on layer-by-layer printing, the landmark innovation of CLIP is to achieve continuous printing in the *z*-axis direction, while resulting in high resolution and high printing speed. Previous studies have shown that CLIP-printed 3D scaffolds containing drug-active molecules show good biocompatibility and biodegradability, and clinically relevant drugs can be loaded and released in a controlled manner [54]. In the future, the advanced rapid printing technology is promising for the development of neural tissue engineering scaffolds.

## 4. 3D Printing Materials for Nerve Regeneration

### 4.1. Hydrogels

#### 4.1.1. Printable Hydrogels

Hydrogel is a very hydrophilic 3D network structure gel, which is widely used in 3D printing [55,56]. When using hydrogels for 3D printing, we usually consider the rheological properties and cross-linking mechanism of hydrogels. Ali et al. [57] obtained the dependence between process parameters (temperature, suction speed, and print head speed) and the physical properties of the hydrogels by testing the effect of temperature on the fluidity of hydrogels (viscosity-temperature curve). The cross-linking mechanisms of hydrogels are physical or chemical. For example, cyclic freezing of polyvinyl alcohol is a kind of physical cross-linking method [58], the ionic cross-linking method between chitosan and tripolyphosphate is also physical cross-linking [59], but usually the physical cross-linked hydrogel is weak in mechanical properties. Chemical cross-linking is mainly used in the manufacture of stable hydrogel structures. It is commonly used to modify the polymer precursors of hydrogels with photopolymerizable functional groups. Wang et al. [60] synthesized acrylated poly (ethylene glycol) -co-poly (xylitol sebacate) copolymers hydrogel 3D printing ink, which can be cross-linked by photopolymerization. According to the source, hydrogels can be divided into natural hydrogels, synthetic hydrogels, and more complex components composite hydrogels [21]. Table 1 summarizes natural, synthetic and composite hydrogels and compares their properties.

#### 4.1.2. Natural Hydrogels

Natural hydrogels are extracted from substances in nature and can be divided into polysaccharide hydrogels (alginate hydrogel, cellulose hydrogel, chitosan hydrogel, heparin hydrogel, hyaluronic acid hydrogel, etc.) and peptides/protein hydrogel (collagen hydrogel, gelatin hydrogel, silk fibroin, etc.) [21]. Many of these can be applied to 3D printed neural scaffolds. This section will discuss the most widely used natural polymer hydrogels in 3D printing.

Alginate, an anionic polysaccharide mainly present in brown alga, is recognized as the most commonly used material in 3D bioprinting due to its non-toxicity and good biocompatibility [59]. Due to the special molecular structure of the guluronic acid block in the alginate, it can cross-link with polyvalent metal ions such as Ca^2+^ into a gel under mild conditions and non-toxic reagents [70,71]. Hashimoto et al. [72] implanted alginate gel between the proximal and distal stumps of sciatic nerve injury in rat, and the regenerated axons were surrounded by Schwann cells to form small bundles after 1~2 weeks. It has a distribution pattern similar to that of normal nerves 21 months after surgery. Compared with implanted collagen sponge and fibrin glue, nerve regeneration in alginate hydrogel is much better. Saman et al. [73] used indirect 3D printing to prepare a sodium alginate hydrogel scaffold that embeds Schwann cells at the expense of gelatin frames. The results of the study showed that Schwann cells in this scaffold were more viable than those in bulk gel.

Chitosan is a polysaccharide obtained by deacetylation of chitin, and its common cross-linking method is ionic cross-linking method, such as by using tripolyphosphate [61]. Chitosan is a low-cost material with good biodegradability and biocompatibility, which has been approved by the FDA (U.S. Food and Drug Administration). In recent years, the application of chitosan in medicine has increased significantly [74]. Li et al. [75] created chitosan conduit with seamless lateral walls and longitudinal arrangement. The highly aligned microstructure can accelerate the directional growth of new tissues, while the porous lateral walls are expected to facilitate the loading of biological factors and reduce nutrient leakage or neuronal extraneous growth. Such conduit can significantly promote sciatic nerve regeneration in rats with a 10 mm gap. Wu et al. [76] reported that complex 3D structures made from chitosan ink can be printed at room temperature. This 3D scaffolds have good mechanical properties and have promising prospects in tissue engineering. Carvalho et al. [77] prepared chitosan nerve conduit with acylated gellan gum as a lumen filler. The metabolic activity of Schwann cells seeded on the hydrogel increased, indicating that this conduit has the potential for neural tissue engineering.

Collagen is a biological macromolecule widely existing in animal tissues. Self-assembled gelation can form collagen hydrogels [78]. In addition, collagen can be cross-linked through dehydration and heating, ultraviolet irradiation, glutaraldehyde cross-linking, etc. [21]. Because of its good biocompatibility, biodegradability and bioactivity, it has been widely used in food, medicine, tissue engineering and other fields [62]. Commercial collagen-based nerve conduits such as NeuroMatrix^®^, Neuroflex^®^(Collagen Matrix Inc. Oakland, NJ, USA) and NeuraGen^®^(Integra Lifesciences Corp. Princeton, NJ, USA) have been approved by the FDA [79]. Bozkurt et al. [80] developed a collagen-based microstructural nerve conduit bridging a 20 mm sciatic nerve defect in rats. Collagen conduit implanted with Schwann cells had a beneficial effect on myelin formation, and the functional recovery of collagen conduit implanted without Schwann cells was as good as that of autologous graft.

Gelatin is an irreversible partial degradation product of collagen after heating. At low temperature, gelatin macromolecules interact with each other to form a solid gel. When the temperature rises, the hydrogen bond weakens, and the structure disperses into liquid state. This property of gelatin can be used to construct scaffolds of various shapes for cell transplantation and tissue regeneration [63]. Tao et al. [81] molded a conduit made of gelatin cryogel through a 3D printed mold at the temperature of −20 °C, which has a porous structure and excellent mechanical properties. It was experimentally evaluated that this conduit can promote the functional recovery of the transverse peripheral nerve after neurological bleeding. However, gelatin dissolves into colloidal sol at normal human body temperature. To solve this problem, the modified gelatin derivative methacrylic acid gelatin (GelMA) was developed and more widely used, which is cross-linked through photoinduced free radical polymerization [82,83]. Billiet et al. [84] printed a mechanically stable cell-filled GelMA scaffold with high cell viability (>97%), which is a potential material for neural tissue engineering.

Silk fibroin, a natural polymer fibrin extracted from silk, has good flexibility and tensile strength, and its non-toxicity and good biocompatibility are suitable for developing into functional materials [64]. Due to its softness, silk fibroin is seldom used to prepare 3D printing scaffolds alone, and it is more often combined with other materials to obtain scaffolds with stronger performance. Zhang et al. [85] printed the chitosan hydrogel scaffold with silk fibroin particles incorporated. Compared with the pure chitosan scaffold, adding silk fibroin led to 5-fold enhanced compressive modulus and improved printing accuracy and stability. Silk loading also adjusted the surface roughness of the scaffold and improved its biodegradability. Jiang et al. [86] printed collagen/silk fibroin scaffolds, which were implanted with rat neural stem cells (NSCs) to facilitate the spinal cord repair in rats.

Except for the frequently applied natural hydrogels above, there are also some other printable natural materials used in neural tissue engineering, such as cellulose. Kuzmenko et al. [87] performed 3D printing by using cellulose nanofibril hydrogels and carbon nanotubes. Cell culture showed that nerve cells prefer attachment, proliferation, and differentiation on 3D printed scaffolds. Generally speaking, natural polymer hydrogels have good biocompatibility, a wide range of sources, and simple methods of obtaining, but they are relatively weak in mechanical properties. Studies have shown that the combination of two different natural hydrogels for 3D printing can be complementary [88]. For example, the combination of gelatin and silk fibroin balances mechanical properties, biocompatibility and degradation rate [89]. More researches have combined natural hydrogels with synthetic hydrogels [28].

#### 4.1.3. Synthetic Hydrogels

Hydrogels composed of synthetic polymers have more flexible mechanical properties and degradation rates compared with natural hydrogels, and they have been applied in various fields of biomedicine [90]. The various synthetic hydrogels suitable for 3D printing neural scaffold manufacture are summarized in Table 2.

Polyvinyl alcohol (PVA) is a water-soluble polymer polymerized by vinyl acetate. The cyclic freezing method is considered to be the best method for preparing PVA hydrogels because it does not involve toxic chemical cross-linking agents [91]. Ulises et al. [65] polymerized PVA hydrogel with biological macromolecules sericin and gelatin (PVA-SG) to encapsulate Schwann cells. The scaffold showed support to cell viability and expression of extracellular matrix proteins. When Schwann cells are encapsulated with PC12, PVA-SG hydrogels support the development of neural networks. Anastasia et al. [92] developed a directed multichannel hydrogel based on chitosan-PVA components that mimic the structures of the epineurium and sheath in nerves. In vitro cytotoxicity tests show the biocompatibility of hydrogels, which is promising for the production of scaffolds that mimic natural peripheral nerve structures. PVA hydrogels have been used in nerve tissue engineering, although not in the form of 3D printing, the 3D-printed PVA scaffolds have been used in some other tissue engineering fields, including cartilage tissue repair [93]. In the future, 3D printed scaffolds of PVA hydrogels will also hopefully be applied to peripheral nerve tissue engineering.

Polyurethane (PU) is a copolymer formed by the polymerization of polyols, small-molecule chain extenders, and isocyanates. The physical and chemical properties of polyurethane can be changed by changing the composition and ratio of hard and soft segments in the molecular chain. Hydrophilic but insoluble polyurethanes can be obtained by adding hydrophilic soft segments (such as polyethylene oxide). PU hydrogels have been used in many biomedical applications [66]. Hsieh et al. [16] synthesized two thermally responsive PUs that form gels around 37 °C without any cross-linking agent. Before gelation, NSCs were embedded in the PU dispersion, and then the dispersion containing NSCs was printed and kept at 37 °C. The NSCs in 25–30% PU2 hydrogel have excellent proliferation and differentiation ability, and the function of adult zebrafish brain injury can be restored after the implantation of 3D printed scaffold. Lin et al. [94] synthesized a new type of thermally responsive waterborne polyurethane hydrogel as a bioink. 3D printed PU hydrogel scaffolds loaded with FoxD3 plasmid (reported to reprogram human fibroblasts into neural rest stem-like cells) and human fibroblasts support human fibroblasts to undergo reprogramming and differentiate into similar neural structure after induction. Huang et al. [95] mixed graphene or graphene oxide with PU to prepare graphene-based nanocomposite hydrogels for NSCs printing. Its rheological properties are suitable for NSCs printing and survival. The printed scaffold significantly enhances oxygen metabolism and differentiation of NSCs.

Polyethylene glycol (PEG), also known as polyethylene oxide (PEO), is one of the most biocompatible and widely used synthetic polymer hydrogels approved by the FDA. Its molecular structure determines that PEG is hydrophilic, and the hydroxy-terminated group can be converted into other functional groups for modification [67,98]. The most common ones are polyethylene glycol diacrylate (PEGDA), polyethylene glycol dimethacrylate (PEGDMA), etc. The cross-linking method of these kind of hydrogels are photopolymerization, so they are suitable inks for SLA. They cannot be naturally degraded, and by adding polyester and other degradable segment chains the degradation rate of PEG hydrogels can be adjusted [56]. Christopher et al. [96] used the photo-curable PEGDA solution as a prepolymer, and used microstereoscopic lithography to prepare nerve scaffolds for in vitro Schwann cell dorsal root ganglion culture. They were able to support reinnervation across the 3 mm injury gap after 21 days, and the results were close to the autograft control. Dilla et al. [97] prepared PEG-polypropylene maleate (PPM) copolymer and PEG-polypropylene fumarate (PPF) copolymer. The hydrogels were printed by DLP to obtain a 3D scaffold with 10 times more elongation at break than the traditional diethyl fumarate (DEF) printing scaffold. In addition, PPF-PEG-PPF triblock hydrogel and primary Schwann cells have been found biocompatible and potential for neural tissue engineering.

#### 4.1.4. Composite Hydrogels

Composite materials consist of two or more constituent materials with markedly different physicochemical properties [99]. Single-component hydrogels are single performance and relatively weak in mechanical strength. Composite hydrogels can combine the advantages of different materials to flexibly design printing inks to meet the mechanical and physiological requirements of host tissues [100]. There are many types of materials that have been used in combination with hydrogels to obtain composite hydrogels, such as particles (including micelles and microspheres), electrospun fibres, nanocellulose, and conductive polymers, etc. [101].

There are already natural-natural hydrogels used for bioprinting nerve scaffolds. Lee et al. [102] prepared collagen-fibroin scaffold. Collagen is selected for the NSCs culture due to its characteristic of supporting cell adhesion and proliferation. Fibroin is chosen to deliver VEGFs due to its ability to act as an affinity-based delivery system. The combination of these two natural hydrogels achieves the dual goal of printing NSCs and VEGF delivery. Huang et al. [88] shows that the high hydrophilicity of alginate hinders the attachment of proteins and cells, which can be solved by peptide modification with Arg-Gly-Asp (RGD). Gelatin retains the RGD sequence, which is less immunogenic and promotes cell adhesion. Therefore, the combination of alginate and gelatin promotes cell adhesion to the surface of the scaffold, increasing the compatibility of the scaffold and Schwann cells. Natural-synthetic hydrogel has the flexibility to optimize the processability of 3D printing. Zhu et al. [28] printed GelMA-PEGDA composite hydrogel scaffold, in which GelMA retains the natural cell binding motif required to support cell adhesion, proliferation, migration, but it has limited mechanical properties. The mechanical properties of PEGDA hydrogel can be fine-tuned by the molecular weight and concentration of PEGDA, as well as the exposure intensity and time. Thus, the combination of PEGDA and GelMA ink created a mechanically strong and suitable cell adhesion composite 3D scaffold by SLA.

Some other functional materials are also used to manufacture composite hydrogels. Jafarkhani et al. [103] synthesized nano graphene oxide/chitosan (NGO/CHT) composite hydrogels, the NGO addition changed the pore structure and improved mechanical strength of hydrogel and increased nerve cells growth up to 20%. More examples of conductive composite hydrogels are introduced in Section 4.3.1. Chen et al. [104] bioprinted multiscale composite scaffolds based on GelMA/chitosan microspheres (GC-MS), and these scaffolds with hydrogel microspheres provided a suiTable 3D microenvironment for enhancing neurite growth. Table 3 compares some composite hydrogels for 3D printing neural scaffold manufacture.

### 4.2. Polyesters

Due to the advantages of simple operation process and low cost, the polyester scaffold manufactured by 3D printing such as FDM is also widely used in neural tissue engineering. There are ester functional groups on the polymer backbone of polyester, such as Polylactic acid (PLA), polyglycolic acid (PGA), polylactic acid-glycolic acid copolymer (PLGA), polycaprolactone (PCL), polyurethane (PU), etc. [106]. Polyester has attracted wide attention in various tissue engineering applications due to its good biocompatibility, acceptable biodegradability and thermoplastic processability [107]. Hydrogel scaffolds are limited in mechanical stability and have a potential risk of swelling. In comparison, polyesters have higher stiffness and stability than hydrogels [108,109].

Polyester ink can be obtained either by melting or by dissolving in organic solvents. The molten polyester can be printed into a 3D scaffold by extrusion. After the raw material is placed in a heating device connected to a metal nozzle, the polyester is melted and extruded. Because of the heat dissipation at room temperature, the extruded pattern quickly solidifies, thus the polyester scaffold can be constructed layer by layer. Polyesters dissolved in organic solvent can be used as ink for extrusion, inkjet and electrohydrodynamic jet (EHD jet), which require fast solvent evaporation or sublimation rate to obtain a 3D scaffold that does not collapse [110,111]. In addition, photopolymerizable polyesters obtained by grafting acrylic group can be used as printing inks based on photopolymerization [50].

PLA, PLGA, and PCL are all biodegradable thermoplastic polyesters with melting points between 50 and 200 °C, which are easily reached by heating. PLGA is the polymerization of two monomers, lactic acid, and glycolic acid. Compared with pure PLA or PGA, PLGA exhibits a wider range of solubility and the degradation rate of PLGA can be adjusted by the ratio of PLA and PGA. However, as they degrade, they produce neurotoxic lactic acid. PCL is easily soluble in organic solvents, but its degradation rate is relatively slow [111,112,113]. The characteristics of these polyesters melted at high temperature or dissolved in volatile or sublimated organic solvents at normal temperature can meet the requirements of 3D printing, the good compatibility between different polyesters also allows two or more polyesters to be combined for printing. Table 4 compares and summarizes polyesters for 3D printing neural scaffold manufacture.

### 4.3. Composites

In addition to the combination of hydrogel and hydrogel, polyester and polyester introduced above, there are many composite scaffolds with new functional materials added or doped with hydrogel or polyester. Not only do composites combine the characteristics of the two materials, but they also show some new or enhanced characteristics (mechanical properties, electrical properties, stimulus response, cell adhesion, etc.) [21,118]. At present, the most widely used materials in neural tissue engineering are conductive polymers and carbon-based nanomaterials.

#### 4.3.1. Conductive Polymers

Conductive polymer materials are widely used in nerve tissue scaffolds because one of the main functions of nerves is the transmission of electrical signals. Electrical stimulation enhances cell proliferation, migration, differentiation, and signaling connections between nerve cells, thereby enhancing neural regeneration and supporting the differentiation of stem cells into nerve cells [119,120]. Polypyrrole (PPy) [121,122], polyaniline (PANI) [123], poly (3,4-ethylenedioxythiophene) (PEDOT) [124,125] are the most promising conductive polymers for tissue engineering [126]. Many researchers indicated that conductive materials show good biocompatibility when co-cultured with cells in vitro [127]. Doping of conductive polymer is currently a widely used method, but the conductive polymer may not be naturally degraded in vivo and produce inflammatory reactions, which can be solved to some extent by combining with biodegradable materials [128]. In addition to doping conductive polymers to prepare conductive hydrogels, synthesis of conductive hydrogel is another kind of method for conductive neural scaffold. Alizadeh et al. [123] synthesized chitosan aniline pentamer and mixed it with alginate and agarose to prepare hydrogels, whose conductivity can reach 10^−3^ s/cm. Olfactory ecto-mesenchymal stem cells (OE-MSCs) were induced to differentiate into dopaminergic neuron-like cells on the hydrogel.

The integration of conductive polymers with hydrogels or polyesters for 3D printing has been applied in many fields, especially biosensors and tissue engineering. The research of nerve regeneration and nerve differentiation by electrical stimulation has progressed greatly. Heo et al. [129] freeze-dried the PEDOT: PSS aqueous solution and mixed it with PEGDA to obtain a photocurable prepolymer and SLA 3D printers were used to pattern conductive hydrogels on substrates. As the concentration of PEDOT: PSS increases, the conductivity of the hydrogel increases significantly. In addition, the integrated GelMA hydrogel wrapped by DRG cells and the 3D printed conductive structure together enhance the efficiency of neural differentiation under electrical stimulation. Fantino et al. [130] 3D printed PEGDA hydrogels and conducted chemical oxidation polymerization of pyrrole to prepare conductive PPy hydrogels. By adjusting the reaction conditions, the PPy can spontaneously permeate into the hydrogels to achieve the desired conductivity. Sanjairaj et al. [131] printed a PCL-PPy nerve conduit by electrohydrodynamic jet 3D printing process. Human embryonic stem cell-derived neural crest stem cells (hESC-NCSCs) were able to attach and differentiate to peripheral neurons on PCL and PCL/PPy scaffolds, which suggests that PPy-based conductive scaffolds have potential clinical value for peripheral neuronal regeneration.

#### 4.3.2. Carbon-Based Nanomaterials

Carbon-based nanomaterials with unique electrical, mechanical, and biological properties have been applied for tissue engineering, of which graphene, single-walled carbon nanotubes (SWCNT), and multi-walled carbon nanotubes (MWCNT) are widely used. Graphene is one of the allotropic forms of carbon, consisting of flat monolayer carbon atoms arranged in a two-dimensional (2D) honeycomb lattice. Each atom has an s-orbital and two in-plane p-orbitals, which contribute to the mechanical stability of the carbon sheet [132]. In recent years the great interest in graphene is mainly due to its unusual properties, including high electron mobility at room temperature, excellent thermal conductivity, excellent mechanical properties, and high specific surface area [133,134]. Park et al. [135] showed that human NSCs on a graphene matrix can differentiate into neurons instead of glial cells. A rolled graphene sheet will form SWCNT, while more than one concentric graphene sheet will form MWCNT [136]. Similar to graphene, CNT also has excellent mechanical strength and high electrical and thermal conductivity.

The combination of carbon-based nanomaterials with hydrogels and polyesters has also been widely used in neural tissue engineering. Metin et al. [137] printed a graphene-added gelatin nerve conduit, where 80% of mesenchymal stem cells showed Schwann cell marker staining and significantly increased the secretion of nerve growth factor (NGF) by applying electrical stimulation to this neural scaffold. Koppes et al. [138] prepared a collagen hydrogel conduit with SWCNT filler. Compared with the control without SWCNT filler, the growth of neurites increased by 3.3 times in the 20 μg/mL SWCNT loaded hydrogel scaffold. The simultaneous presence of electrical stimulation and the SWCNT-loaded hydrogel scaffold resulted in a 7.0-fold increase in neurite outgrowth, suggesting that the SWCNT filler scaffold and electrical stimulation may have a synergistic effect in promoting neurite outgrowth. Jakus et al. [139] obtained a conductive composite scaffold by extruding graphene PLGA ink, which showed high mechanical strength and flexibility, while maintaining a conductivity greater than 800 S/m. In vitro experiments showed that the composite scaffold supported the adhesion, proliferation, and neurogenic differentiation of human mesenchymal stem cells (hMSCs), and significantly upregulated glia and neuronal genes. Qian et al. [114] printed PCL and single-layer graphene or multi-layer graphene composite scaffold and polydopamine (PDA) and arginyl glucosyl aspartate (RGD), which can improve cell adhesion and are coated on the surface of graphene. This conductive 3D scaffold can significantly improve nerve expression in vitro and in vivo. Table 5 compares and summarizes 3D printed neural scaffolds with conductive materials or carbon-based nanomaterials added.

## 5. 3D Bioprinting and Therapeutics Delivery for Nerve Regeneration

3D bioprinting means printing biomaterials or cell units according to the principle of additive manufacturing driven by digital 3D models [141]. The bioink used for bioprinting may include cells, bioactive components and/or biomaterials [142]. Considering cell viability and biomolecule activity, the conditions of 3D bioprinting are more severe than ordinary 3D printing. First, bioprinting should keep the printing fidelity and cell viability at the same time, so the chemical and biological properties of viscosity, shear force, gel speed, and cell compatibility will affect the application of bioink. Secondly, the selection of biomaterials also affects cell adhesion, growth, proliferation, and even differentiation because the interaction between cells and matrix affects the direction of cell growth or differentiation [40]. In addition to the three printing methods based on inkjet, extrusion, and SLA introduced in the previous section, there are some special printing methods developed for bioink. This section will discuss bioprinting methods, cells, bioactive molecules, and drug delivery in neural tissue engineering.

### 5.1. Bioprinting Methods

Extrusion-based printing is one of the most widely used technologies to constructing 3D structure embedding cells for tissue engineering. The compressive force and shear stress generated by extrusion are the main causes of cell damage, so the printing parameters (cell density, bioink viscosity, temperature, air pressure, etc.) need to be optimized to prevent cell apoptosis [56]. The biggest advantage of this bioprinting method is that it can print viscous bioinks with high cell density, which cannot be easily achieved by inkjet and SLA. On the other hand, compared with the other two printing methods, the resolution of extrusion bioprinting is slightly inferior, and it has relatively low ability to accurately preset the printing shape and fixed cell position. Hsieh et al. [16] constructed a neural scaffold with thermally responsive PU ink loaded with NSCs by FDM. The mixture of NSCs and PU is printed in a pile in the form of stacked fibers through a 250 μm nozzle, and the fibers can be stacked into eight layers (about 1.5 mm thick) without serious collapse.

The inkjet technology for bioprinting has successfully printed liquid bioink. In order to prevent nozzle clogging, the ink must exhibit low viscosity and low cell density. The control of these conditions in turn causes other limitations in the printing process (diffusion of droplets, cell sedimentation, etc.). However, inkjet has advantages in resolution, and studies have shown that the precise positioning of cells by inkjet can promote the establishment of fine neuronal networks [56]. Tae et al. [143] used a piezoelectric inkjet printer to print porcine Schwann cells and neuronal analogs NG108-15 cells, and detected neuron and glial cell viability of >86% and >90%. The printed neuronal cells produced longer neurites earlier than the control group. Compared with standard cell seeding, there was no significant difference in cell viability. This result provides a broad platform for nerve regeneration methods. Lee et al. [102] bioprinted murine NSCs, collagen hydrogel and fibrin gel releasing VEGF by inkjet to construct artificial nerve tissue. The printed cells showed a high survival rate and the scaffold can induce NSCs morphology changes and migration.

SLA is one of the most promising bioprinting methods because of its high resolution and ability to manufacture scaffolds containing high cell density. Compared with extrusion and inkjet, there is no nozzle clogging in SLA, which expands the range of bioink viscosity and cell density. However, the basis of SLA is that bioink should be compatible with laser wavelength, and therefore cell viability may be affected [144].

Bioplotting is a biological printing method based on extrusion. Unlike FDM, the extrusion head is moved by the computer in the three directions of x, y, and z axes, and the production platform remains stationary [145]. After extrusion, the material is solidified in some way, such as ionic cross-linking or photopolymerization. Rajaram et al. [146] printed alginate/hyaluronic acid (HA)/Schwann cells by bioplotting. Each scaffold is immersed in a calcium chloride bath for cross-linking, which maintains the scaffold structure while maintaining the activity of Schwann cells. Porous alginate/HA scaffolds with good structural integrity and long-term cell viability is suitable for neural tissue engineering. Chen et al. [104] used 3D-Bioplotter to extrude GelMA hydrogel ink loaded with NGF chitosan microspheres at a speed of 2 or 5 mm/s, and then solidified the scaffold under 365 nm ultraviolet radiation. The PC12 cells and Schwann cells wrapped in the scaffold showed considerable cell viability compared to unprinted cells.

**Figure 5:**
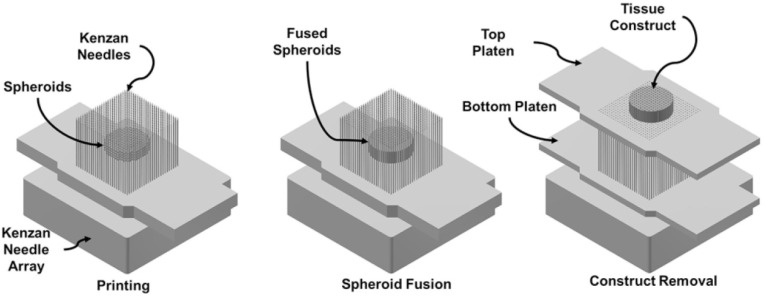
Schematic illustration of Kenzan method. Reproduced with permission [150]. Copyright 2019, Elsevier Ltd.

The Kenzan method is a technology that directly assembles cells without the aid of other materials [147]. Kenzan means “needle array” in Japanese. Firstly, cell aggregates of single or mixed cell types are cultured in vitro. According to the pre-designed 3D data, the spherical cell aggregates are placed in an array composed of fine needles. There is contact between adjacent spheres to obtain a closely arranged 3D shape, which is transferred to a bioreactor and cultivated to obtain an ideal tissue (Figure 5). In the Kenzan method, it is the contact between cells that supports the 3D structure, rather than materials such as hydrogels used in traditional 3D printing, so Kenzan is also called scaffold-free bioprinting [148]. At present, implementation of the Kenzan method mainly relies on Regenova printers. Zhang et al. [149] demonstrated that human gum-derived mesenchymal stem cells (GMSCs) have a tendency to aggregate into 3D spheroids, and that spheroids GMSCs are more likely to differentiate into neurons and Schwann-like cells than adherent ones. The Regenova printing system was used to print the spherical GMSCs and make them mature in the bioreactor. The in vivo transplantation of the neural structure carrying GMSCs promoted the regeneration and functional recovery of facial nerve injury in rats.

### 5.2. Cells and Bioactive Molecules

Biologics such as cells and growth factors play important roles in peripheral nerve regeneration [151,152]. After peripheral nerve injury, Schwann cells will form a Büngner band to guide the regeneration of axons from the proximal stump to the distal stump. While they secrete NGF and promote axonal regeneration and remyelination. It is important to establish the interaction between Schwann cells and axons as soon as possible after injury [153]. Patient-derived stem cells, such as NSC, induced pluripotent stem cell (iPSC), GMSC, bone mesenchymal stem cell (BMSC), have the potential to differentiate into cells for nerve regeneration [154]. Biologically active molecules have a strong effect on nerve regeneration, such as NGF, brain derived neurotrophic factor (BDNF), ciliary neurotrophic factor (CNTF) [155], VEGF [156]. For example, NGF is implicated in both cell survival and guidance of regenerating nerve tissue [157,158]. Table 6 describes the cells and bioactive molecules that have been bioprinted in neural tissue engineering.

There are many factors that affect the cell viability in bioprinting. Firstly, the biocompatibility of materials, as natural materials with good cellular compatibility are always selected as carriers for bioprinting, and synthetic materials are used to combine with natural materials to make up for the lack of mechanical properties. Moreover, the degree of cross-linking of the material and the cytotoxicity of the cross-linking agent, by-products, and initiators also need to be considered. Second, the cell density of the bioink influence, because if the density is too low, the cell growth and proliferation rate may be affected, making it difficult to achieve the ideal nerve repair. When the density is too high, the high mechanical force and high shear force generated by high pressure may damage the cells, thereby affecting cell viability. It can be seen from Table 6 that the cell density in most bioinks is controlled within the range 10^5^~10^7^ cells/mL.

### 5.3. Therapeutics Delivery

In addition to cells and biologically active molecules, drugs used to repair nerve injury can also be loaded in nerve scaffolds. Manoukian et al. [160] manufactured a nerve conduit with a voltage-gated potassium channel blocker 4-Aminopyridine (4 AP), which is a growth factor replacement that can enhance the rate of nerve regeneration. It has been found to prolong nerve action potential and strongly promote the release of neurotransmitters due to its potassium channel blocking function, which can increase the speed and degree of functional recovery of mild nerve crush injury [161]. The nerve conduit with 4 AP was co-cultured with Schwann cells. In the first seven days, the conduit released a total of 30 ± 2% of the encapsulated 4 AP, which promoted the expression of both neurotrophic factors and brain-derived neurotrophic factors and guided the axon regeneration.

There are many similar drugs that affect nerve regeneration through different mechanisms, some of which have been added to 3D printing inks to build neural scaffolds that can deliver and control the release of drugs. In recent years, some drugs have been encapsulated in nanoparticles and then loaded into 3D printed scaffolds. This method not only ensures the integrity and effectiveness of drugs, but also extends the drug release time. Xu et al. [162] loaded the drug RGFP966 into MPEG-PCL nanoparticles, and then dispersed the nanoparticles in GelMA to construct a drug-loaded 3D neural scaffold through DLP bioprinter. The activity of histone deacetylase 3 (HDAC3) is neurotoxic, which becomes a negative regulator of chromatin modification by inhibiting the activation of the PI3K–AKT–ERK signaling pathway. In contrast, activation of PI3K–AKT and ERK signaling pathways leads to the occurrence and regeneration of peripheral myelin sheaths. Therefore, the HDAC3 inhibitor RGFP966 can promote myelination by targeting the PI3K-AKT-ERK signaling pathway. XMU-MP-1 (Hippo pathway inhibitor), which was also encapsulated in a similar way, facilitates the yes-associated-proteins (YAP) high expression in the nucleus, thereby promoting the interaction between YAP and the myelin gene promoter and controlling the extension of myelin [163]. The drug-loaded 3D printed GelMA-PEGDA scaffold effectively induced the recovery of sciatic nerve injury, showing potential clinical applications in peripheral nerve repair. This drug-loaded 3D printing method can be extended to many similar drug molecules that affect peripheral nerve regeneration and has a broad space for further development and exploration.

## 6. Summary and Prospect

The ideal neural scaffold requires not only reliable mechanical properties but also an elaborate guiding structure, which is well addressed by the highly controlled flexible 3D printing methods. 3D printed neural scaffolds have a history spanning decades, and the recent advances are now reflected in: (1) various natural and synthetic hydrogels and polyester being developed with tailorable mechanical physical, chemical and biological properties as 3D printing inks to match the requirement of nerve regeneration; (2) conductive polymers and carbon-based nanomaterials are used in 3D printing to obtain nerve scaffolds with excellent performance; (3) new 3D printing techniques and/or instruments will be explored to fabricate a high-precision neural scaffold while solving the problem of printing speed such as TPP and CLIP; (4) stem cells from many different sources are bioprinted into neural scaffolds to induce differentiation into neural cells; (5) drugs that affect nerve regeneration through different mechanisms are used in 3D printed scaffolds to play a role similar to growth factors.

3D printing technologies are leading to customized nerve therapies and drug delivery systems, which holds great promise for patient-specific healthcare, personalized medicine and precision medicine. However, there are still many possibilities in this area waiting for our development. In order to achieve smart changes in response to the host’s internal environment, 4D printed neural scaffolds may be developed. 4D printing is regarded as a further development of 3D printing. This concept was first proposed by Skylar Tibbits in a TED speech in 2013 [164], and researchers have been inspired by it. The structure of 3D printing has always been fixed and static by default, but 4D printing extends 3D printing to another one dimension. The dynamic change in time and space related to the 3D printing results is the fourth dimension. The change comes from the stimulating factor, including temperature, pH, light stimulation, electrical stimulation, etc. Due to the characteristics of smart materials, printed scaffolds can simulate the dynamic response of tissues to stimuli. When applying electrical stimulation to the 3D scaffold, it not only affects the growth and migration of nerve cells [130], but also affects the differentiation of stem cells into nerve cells. Metin et al. [137] reported that the electrical stimulation applied to the 3D gelatin graphene scaffold played a positive role in the differentiation of mesenchymal stem cells into Schwann cells. Eighty percent of the cells showed Schwann cell marker staining and significantly enhanced NGF secretion. Similarly, low-level laser stimulation can also achieve the purpose of promoting the differentiation of stem cells neurons. Zhu et al. [165] printed the GelMA/PEGDA hydrogel 3D transparent scaffold and seeded the NSCs on the scaffold while applying 635 nm low light stimulation. The results showed that this light stimulation can promote the differentiation of NSCs. At present, in terms of neural tissue engineering, 4D printing is mainly oriented to the response of the cells carried by the scaffold to external stimuli, and there are few studies on the changes of the scaffold itself. In the future, 4D printed nerve conduit may have shape memory function. After being implanted in human nerves, joints and other places, it can self-adjust with the bending or stretching movement to achieve a longer service life. The possibilities in this area are still waiting for us to explore.

## Figures and Tables

**Table 1:** Comparison and summary of hydrogels.

Materials	Advantages	Disadvantages
Natural hydrogels (alginate hydrogel [59], chitosan hydrogenl [61], collagen hydrogel [62], gelatin hydrogel [63], silk hydrogel [64] etc.)	Low inflammation; wide variety of sources; good biodegradability and biocompatibility	Poor mechanical properties
Synthetic hydrogels (PVA hydrogel [65], PU hydrogel [66], PEG hydrogel [67], PAM hydrogel [68] etc.)	Tunable mechanical properties, degradation rate and biocompatibility; good durability	Possible chronic inflammation
Composite hydrogels [69]	Combining the characteristics of different materials; Flexible optimization for the processability of bioprinting	Limitations in homogeneous ink preparation

**Table 2:** Synthetic hydrogels for 3D printing neural scaffold manufacture.

Material	Printing Resolution	Printed Structure	Mechanical Properties	Printing Method	Reference
PU	250 μm	Grid scaffold of stacking fibers	-	FDM	[16]
PU	410 μm	Grid scaffold of stacking fibers	-	Extrusion	[94]
PU	410 μm	Grid scaffold of stacking fibers	-	Extrusion	[95]
PEGDA	50 μm	guidance Conduits with trenches	Young’s modulus of 470.0 ± 24.3 MPa	SLA	[96]
PEG-PPF	100 μm	Gyroidal scaffold	Young’s modulus of 9.1 ± 0.1 kPa	DLP	[97]

**Table 3:** Composite hydrogels for 3D printing neural scaffold manufacture.

Components	Printing Resolution	Printed Structure	Mechanical Properties	Printing Method	Reference
Collagen-fibroin hydrogel	500 μm	Double-layered 3D scaffold	-	Inkjet	[102]
Gelatin-alginate hydrogel	160 μm	Scaffold with void channel	-	Extrusion	[88]
PEG-PEGDA hydrogel	300 μm	Grid scaffold of stacking fibers	Young’s modulus of 1.01 ± 0.11 MPa	SLA	[105]
GelMA-PEGDA hydrogel	2.5 μm	Guidance conduit with four microchannels	Young’s modulus of 0.3~4.5 MPa	DLP	[28]
GelMA/GC-MS hydrogel	~545 ± 61μm	Grid scaffold of stacking fibers	-	Extrusion	[104]

**Table 4:** Polyesters for 3D printing neural scaffold manufacture.

Materials	Printing Resolution	Printed Structure	Mechanical Properties	Printing Method	Application	Reference
PLA	250 μm	Scaffolds with different gap width between struts	-	Extrusion	Promotes neural differentiation of hDPSCs.	[39]
PCL	50 μm	Porous guidance conduit	Elastic modulus of 68.74 MPa	Inkjet	Promotes successful axonal regrowth and remyelination	[114]
PLA/PCL	1 μm	Guidance conduit	-	Inkjet	ability to sustain cell growth and attachment	[38]
PCL/PAA	~50 μm	Grid porous conduit	Young’s modulus of 85 ± 3.9~204 ± 6.7 MPa	EHD jet	Influence nerve excitation and conduction	[115]
PU/collagen	~150 μm	Double layer porous conduit	-	Extrusion	Bridge a 10 mm long rat peroneal nerve defect	[116]
PLGA/PLLA	-	Scaffold with guidance channels	Young’s modulus of 2~62 MPa	FDM	Guide axons to linear conformations and support growth of iPSC-derived neurons	[117]

**Table 5:** Composites for 3D printing neural scaffold manufacture.

Conductive Materials	Main Materials	Printing Resolution (μm)	Printed Structure	Mechanical Property (MPa)	Conductivity (mS/m)	Printing Method	Reference
PEDOT	GelMA and PEGDA hydrogel	200	Grid scaffold of stacking fibers	Compression stiffness 26.3–35.4	1510	SLA	[129]
PPy	PEGDA hydrogel	200	Honeycomb structure	Young’s modulus 1.4	7.7	SLA	[130]
Graphene	Gelatin hydrogel/PLA	~100	Porous conduit	Young’s modulus~80	0.02	Extrusion	[137]
Graphene	PLGA	100	Square pore scaffolds	Young’s modulus 3~16		Extrusion	[139]
MWCNT	PEGDA hydrogel	~200	Square pore scaffolds	Young’s modulus ~1.1	~0.08	SLA	[140]
Graphene	PCL	50	Porous conduit	Elastic modulus 68.74	890	Inkjet	[114]

**Table 6:** Bioprinted cells and bioactive molecules for nerve tissue engineering.

Cell	Bioactive Molecule	Material	Printing Resolution	Cell Density	Cell Viability	Printing Method	Application	Reference
Schwann cells	-	Alginate/HA	353 ± 7 μm	5.2 × 10^5^ cells/mL	92.3%	Bioplotting	Good structural integrity and long-term cell viability	[146]
PC12 cell; RSC96 cell	NGF	GelMA/chitosan	545 ± 61 μm	-	97.1 ± 3.69%	Bioplotting	Enhance 3D neurite outgrowth and elongation	[104]
Schwann cells; NG108-15 cells	-	-	60 μm	2 × 10^5^ cells/mL	89–92%; 86–96%	Inkjet	Neurite outgrows faster and earlier	[143]
Schwann cells	-	Gelatin/ sodium alginate	160 μm	2 × 10^6^ cells/mL	91.87 ± 0.55%	Extrusion	Improve cell adhesion and related factor expression	[88]
NSCs	-	PU hydrogel	250 μm	4 × 10^6^ cells/mL	~80%	FDM	Promote the recovery of traumatic brain injury in zebrafish	[16]
NSCs	VEGF	Collagen hydrogel/fibroin gel	700 μm	1 × 10^6^ cells/mL	93.23 ± 3.77%	Inkjet	Support cellular proliferation and migration over time	[102]
Human fibroblasts	Forkhead box D3	PU hydrogel	410 μm	1 × 10^6^ cells/mL	65%	Extrusion	Human fibroblasts could be reprogrammed into neural crest stem-like cells	[94]
NSCs	-	Graphene/PU hydrogel	410 μm	4 × 10^6^ cells/mL	>60%	Extrusion	NSCs had a tendency to differentiate toward glial and neuronal lineages	[95]
iPSCs	-	GelMA/gelatin/fibroin gel	200 μm	1 × 10^7^ cells/mL	>75%	Extrusion	Differentiate and extend axons throughout microscale scaffold channels	[159]
GMSCs	-	-	400~500 µm	-	~90%	Kenzan method	Promote rat facial nerve regeneration	[149]
